# The eusocial non-code: Unveiling the impact of noncoding RNAs on Hymenoptera eusocial evolution

**DOI:** 10.1016/j.ncrna.2024.10.007

**Published:** 2024-10-28

**Authors:** Egor Lebedev, Daniil Smutin, Pavel Timkin, Danil Kotelnikov, Amir Taldaev, Nick Panushev, Leonid Adonin

**Affiliations:** a Institute of Environmental and Agricultural Biology (X-BIO), Tyumen State University, 625003, Tyumen, Russia; b Faculty of Information Technology and Programming, ITMO University, St.-Petersburg, 197101, Russia; c All-russian Research Institute of Soybean, 675027, Blagoveschensk, Russia; d Institute of Biomedical Chemistry, Moscow, 119121, Russia; e Research Center for Molecular Mechanisms of Aging and Age-Related Diseases, Moscow Institute of Physics and Technology, Dolgoprudny, 141700, Russia; f Bioinformatics Institute, 197342, St.-Petersburg, Russia; g Federal State Budget-Financed Educational Institution of Higher Education The Bonch-Bruevich Saint Petersburg State University of Telecommunications, Saint-Petersburg, 193232, Russia

**Keywords:** Hymenoptera, Honey bee *Apis mellifera*, Noncoding RNA, Repetitive DNA, Transposons, Eusociality

## Abstract

Eusociality, characterized by reproductive division of labor, cooperative brood care, and multi-generational cohabitation, represents a pinnacle of complex social evolution, most notably manifested within the Hymenoptera order including bees, ants, and wasps. The molecular underpinnings underlying these sophisticated social structures remain an enigma, with noncoding RNAs (ncRNAs) emerging as crucial regulatory players. This article delves into the roles of ncRNAs in exerting epigenetic control during the development and maintenance of Hymenopteran eusociality. We consolidate current findings on various classes of ncRNAs, underscoring their influence on gene expression regulation pertinent to caste differentiation, developmental plasticity, and behavioral modulation. Evidence is explored supporting the hypothesis that ncRNAs contribute to epigenetic landscapes fostering eusocial traits through genomic regulation. They are likely to play an important role in eusociality “point of no return”. Critical analysis is provided on the functional insights garnered from ncRNA profiles correlated with caste-specific phenotypes, specifical for phylogenetic branches and transitional sociality models, drawing from comparative genomics and transcriptomics studies. Overall, ncRNA provides a missed understanding of both “genetic toolkit” and “unique genes” hypotheses of eusociality development. Moreover, it points to gaps in current knowledge, advocating for integrative approaches combining genomics, proteomics, and epigenetics to decipher the complexity of eusociality. Understanding the ncRNA contributions offers not only a window into the molecular intricacies of Hymenoptera sociality but also extends our comprehension of how complex biological systems evolve and function.

## 1 Introduction

The phenomenon of eusociality is characterized by the presence of distinct castes within a species, with significant examples of its emergence seen in the insect order Hymenoptera, which includes ants, bees, and wasps [1]. Eusocial behavior has independently evolved at least 11 times within this class [2], with bees showcasing six separate instances [[3], [4], [5], [6]], and standalone occurrences within all ants and some wasps [7,8]. These evolutionary transitions represent a critical development and are one of the key factors in the ecological dominance of eusocial Hymenoptera [9].

The evolution of eusociality in insects has been traditionally associated with haplodiploidy, a genetic system that can enhance relatedness among individuals through kin selection [10], but it is not an obligatory mechanism [11]. While phylogenetic analysis has shown a strong correlation between eusociality and haplodiploidy in hexapods [12], there are exceptions like aphids and termites that display eusocial behavior without this genetic mechanism and uncleared molecular basis [6,13,14]. Furthermore, not all Hymenoptera are eusocial. Moreover, there are transitional and reversion to solitary behavior forms [[15], [16], [17], [18]], indicating that eusociality is not an “evolutionarily stable strategy”.

The genetic basis of eusocial behavior remains unclear [2,5,10], with the concept of a "point of no return" [19] in eusocial evolution suggesting a critical juncture where irreversible changes occur at the molecular level [20]. Transcriptome analyses have identified gene networks involved in regulating reproductive division of labor between castes in eusocial species [21,22]. Major differences between castes, as well as between eusocial and solitary species are related to differential expression of regulatory gene networks convergently in different branches [[21], [22], [23]]. More specifically, non-coding RNA (ncRNA) playing a role in gene regulation have been previously hypothesized [20] and identified to facilitate the eusocial transition through shifts in gene expression [20,21,[24], [25], [26]], including influence on the omics networks [[27], [28], [29]]. Differences in the evolution [30,31] and expression [[32], [33], [34]] of non-coding regions between eusocial and solitary species, if not their function, at least their role as markers.

Despite challenges in experimental validation due to phylogenetic influences, identifying common mechanisms underlying independently evolved eusocial organisms may shed light on the essential changes driving eusocial evolution. This review aims to explore these regulatory ncRNA as potential keys to understanding the evolution of eusociality.

## 2 Understanding eusociality

Eusociality is a complex form of social organization observed in various animal species, characterized by reproductive division of labor, cooperative brood care, and overlapping generations within a colony. In addition to regular sociality, eusociality is a hierarchical social structure characterized by distinct castes and a reproductive division of labor [[35], [36], [37]]. Queens are responsible for reproduction, while workers perform tasks such as foraging, nest maintenance, and brood care. This division of labor is associated with high levels of cooperation, task specialization, and altruistic behavior [[38], [39], [40], [41], [42], [43]].

It is worth mentioning that eusociality is an evolving category. It is influenced by a number of factors such as reproductive division of labor, age polyethism, and genetic relatedness among individuals [42]. Studies have shown that eusocial species can exhibit a range of traits and behaviors. For example, *Ropalidia marginata*, a primitively eusocial wasp, has distinct traits such as a docile queen and decentralized work regulation, making it unique among primitively eusocial wasps [44]. In contrast, the ectatommine ant *Rhytidoponera metallica* forms queenless colonies with low intra-colony relatedness, suggesting a transient form of eusociality [45].

The most recent stage in the transition from sociality to eusociality is the emergence of non-reproductive castes, so many articles focus on this mechanism. Comparative genomics show that reproductive division of labor and worker polymorphism influence selection intensity across the genome [46]. Moreover, they indicate molecular parallelism in eusociality development, suggesting a common “genetic toolkit” for caste differentiation at different levels of social complexity [[47], [48], [49], [50], [51]].

Eusociality origins are subjects of considerable debate among researchers. Various theories have been proposed to explain the evolution of eusocial behavior, including inclusive fitness theory, kin selection, and group selection [52]. Hamilton's inclusive fitness theory proposes that eusociality evolves due to the increased inclusive fitness of individuals who assist in the reproduction of closely related individuals [53]. Kin selection, a form of natural selection, suggests that genes promoting altruistic behavior can increase in frequency if they enhance the reproductive success of genetically related individuals [54].

Selective pressures, such as ecological constraints, resource availability, and major transitions in evolution, contribute to the evolution of eusociality. For example, limited resources and high competition can favor the evolution of eusocial colonies as a means of maximizing resource utilization and colony survival [55]. The emergence and persistence of eusociality can be shaped by major evolutionary transitions, such as the evolution of complex social behavior from simpler forms of social organization [56,57]. Finally, eusociality is always associated with living in a high-density population, resulting in differences of the individual and forming of the social immunity mechanisms due to fundamentally different interactions with pathogens [58,59].

While these hypotheses focus on the development of society, they also require a molecular basis. Genomic regulation is an important factor in the development and maintenance of eusociality. Studies have revealed specific gene regulatory networks and epigenetic modifications associated with caste determination, behavioral plasticity, and division of labor in eusocial organisms [32,48,49,60].

There are two sets of eusociality genetic evidence hypotheses [15]. The first is assumed presence of the common"genetic toolkit" [61] to eusociality that controls the "social pathways" [62] responsible for the uniform "ground plan" [63,64] phenotype. Although at first glance similar rather than homologous changes are expected in the case of molecular convergent evolution, these hypotheses are supported by a multitude of studies. The opposite hypotheses suggest mechanisms of eusociality involving different molecular pathways in different groups [65,66]. Both hypotheses may be complementary when compared at different levels of classification. Non-coding RNA via influencing extensive pathways are the keys in proving both these hypothesis sets, which is discussed further.

## 3 Research ways

The study of eusociality molecular basis includes fundamentally different approaches. In the first one, omics networks of phylogenetically similar groups are compared [3,6,8,51]. However, in such a case, a significant proportion of differentially expressed gene groups may show differences in the ecology and phylogeny of the groups. Ants represent a distinct clade with once occurring eusociality, so any genetic mechanism related to it should be consistent. Among wasp and bees eusociality emerged many times, so different convergent mechanisms of eusociality evolution may be found, and it is better to search through similar regulatory pathways. The other approach involves comparing different castes of eusocial organisms [1,24,27,67,68]. Differences in expression patterns may not explain much of the differences in behavior in this case. On the other hand, such differences, identified and confirmed *in natura*, will be real markers of eusociality. Finally, transitions [51,[69], [70], [71]] and deviations [[72], [73], [74]] of eusocial behavior are very interesting models. However, there are few such models with their own specifics, which are more clearly observed at the level of differential expression than major eusocial transition patterns. Finding similar mechanisms by different methods in different groups is a key to confirm the role of mechanism in eusociality.

Otherwise, differences can be sought at different omics levels. Total transcriptomes will always show significant differences, reflecting differences in physiology between stages - not only their causes, but also many of their consequences [32,48,75,76]. Brain omics studies partially solve this problem, while removing possible physiological underpinnings [[77], [78], [79], [80], [81]]. Abdominal RNAseq are more similar among social species [16].

Some studies focus on specific functional mechanisms that involve specific classes of RNAs - coding (especially related to pheromones [33,82]), and various non-coding, claimed to be major expression regulators [30,48]: miRNAs [31,68,83–85], lncRNAs [26,56,78], and others. Comparisons of studies of the coding part of the transcriptome show that there is no "eusociality gene", but there are convergent “eusociality processes” [20,24,29,67]. While closely related groups showed conserved gene duplications, gene family expansions, and the presence of eusociality-associated genes [86], phylogenetically distant groups show molecular convergence mostly on the network level [24,87]. Additionally, transposons have been implicated in the evolution of eusociality, potentially through shifts in gene expression [88]. Transcriptomic analyses have identified molecular signatures associated with caste determination, social behavior, and communication, through the identification of differentially expressed pathways under ncRNA control [21,29,89]. One of the common mechanisms is the queen pheromones trigger of gene expression and epigenetic marker changes, influencing caste differentiation and reproductive behavior in social insects [33,46,82,90,91].

Epigenetic modifications, such as DNA methylation and histone modifications, can influence gene expression and play crucial roles in regulating social behavior. Studies on honey bees, ants, and wasps have demonstrated dynamic changes in methylation patterns associated with caste differentiation and behavioral plasticity [86,92]. Interestingly, DNA methylation in a nutshell was reported not influencing gene expression of honey bees [93]. So, it might indicate the role of other mechanisms. Modulating histone modifications has also been linked to regulating gene expression involved in the development and maintenance of eusociality [94]. These processes also claimed to be under non-coding genome control [20,27,68,95].

Different ncRNA exert different genomic control and require different researching methods. Since the key method of comparison is to compare transcriptomes at the pupal or imago stages, very little information is available on the regulation of embryonic development, which obviously affects eusociality. Thus, it is very difficult to assess the role of developmental piRNA [96,97]. In addition, transcriptomes often provide a truncated view of lncRNAs [98]. The role of miRNAs has been the most studied, also because of the simplicity of such studies [99]. Finally, proving paralogy for any non-coding RNAs is a very difficult task.

## 4 Noncoding RNAs: types and functions

**Fig. 1:**
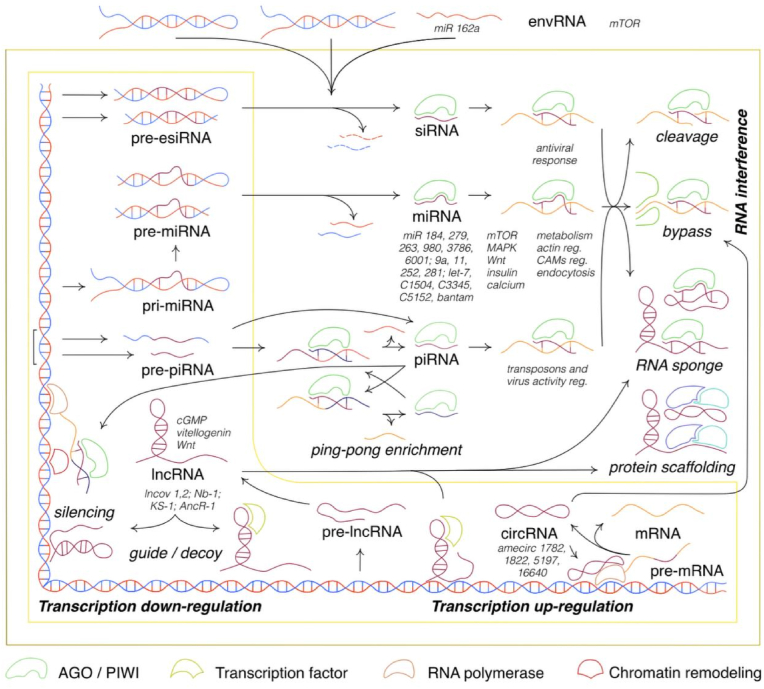
Regulatory ncRNA, their pathways and molecular target links to eusociality. Different types of non-coding RNAs with possible mechanisms of actions are presented. Color and shape legend corresponds to different molecules. Key molecules and the general mechanisms involved in their function are signed in large print. The names of specific non-coding RNAs that play a role in eusociality and the mechanisms of action known for them are labeled in smaller type. All non-coding RNAs are categorized into small and long ncRNAs. Interfering small RNAs (sRNAs) are categorized into small interfering (siRNA), micro - (miRNA) and PIWI-interacting (piRNA) RNAs. Endogenous siRNAs (esiRNAs) are formed from double-stranded pre-siRNA precursors. Typically, miRNA development involves mismatch-containing pri-miRNA and pre-miRNA. Pre-piRNA are single-chain molecules typically produced from a consistent loci (PIWI-box) with possible direct processing to piRNA. Viral piRNA (vpiRNA) is transcribed from endogenous viral elements (EVE). (V)piRNA cytoplasmic pool can be enriched if complementary molecules appear as a result of the "ping-pong pathway". Environmental RNAs (envRNAs) often play (pre-)sRNA roles. Most insect sRNAs interact with Ago proteins during RNA-induced silencing complex (RISC) formation, while piRNAs bind with PIWI. RNA interference (RNAi) could appear via target cleavage or via translation inhibition by tight binding known as bypass mechanism. MiRNAs and siRNAs often bind with mRNAs or long non-coding RNAs (lncRNA), while (v)piRNA targets are transposon and viral RNAs. Mainly piRNA binds with their target in the nucleus during transcription, and recruits chromatin remodeling complexes. All sRNAs are target expression inhibitors. LncRNAs can be diversely processed. By directly guiding target DNA, lncRNA can inhibit or activate genome expression both *cys-* and *trans-*. They can serve as linkers for transcription factors (TFs), activating the expression or repressing it through decoying of TFs. By targeting multiple molecules lncRNA can play a role in their scaffolding or bind with RISCs, acting as a sRNA sponges. Circular RNAs are splicing mRNA products via different mechanisms. Mostly, functional circRNAs and mRNAs cannot be derived from the same molecule, so they have different expression patterns. Often circRNAs activate RNA polymerase and their own gene expression. As lncRNAs, circRNAs could act as protein scaffolders and RNA sponges. Nevertheless, ncRNA scaffolding and circRNA translation are predicted but not described among Hymenoptera. Both circRNA and lncRNA operate as both target expression activators or inhibitors. Elaboration of these concepts is provided in the accompanying text.

Non-coding RNAs (ncRNAs) are a category of untranslated DNA transcripts that play a role in regulating gene expression and maintaining genomic stability. As regulators, ncRNAs have a direct impact on eukaryotic development and physiology. NcRNAs exhibit diversity in size and function, leading to their classification into two families: housekeeping ncRNAs and regulatory ncRNAs (Fig. 1) [100].

All the housekeeping ncRNAs are constitutive components of each eukaryotic cell, whose expression, as above, is crucial for realization of major cellular programs. Despite the obvious participation of housekeeping ncRNAs in gene expression processes in eukaryotes, researchers have not yet found evidence of similar mechanisms in Hymenoptera eusociality. Nevertheless, this does not exclude the possibility of the existence of such mechanisms in this species in principle. Further in-depth study of the noncoding component of the transcriptome will shed light on this question [101].

Regulatory ncRNAs mainly operate as governors of gene expression in epigenetic, transcriptional and post-transcriptional processes. LncRNA and circRNA are also involved in post-translational protein scaffolding. Despite the fact that the regulatory role of housekeeping and regulatory ncRNAs in maintaining genome stability is similar, the cornerstone of their distinguishing is the ability to affect molecular processes on epigenetic and transcriptional level. There are several mechanisms of how they are synthesized and act (Fig. 1).

### 4.1 MiRNA

MiRNA are encoded by discrete genes (21–23 bp) or gene clusters (up to ∼92 bp) and transcribed by RNA polymerase II into pri-miRNA molecules. Then it is processed to pre-miRNA and after cleavage and weaving binds with Ago proteins, forming RISC [102,103]. MiRNA controls target mRNA via RNAi by target cleavage, and rarely - by ribosome bypass. This is countered by differential expression and processing of the miRNAs themselves, as well as the operation of small RNA "sponges" - complementary to lncRNAs and circRNAs (Fig. 1). It was reported that functionally miRNAs play a pivotal role in odor learning, neurogenesis and memory formation [104], reproduction and labor division in social insects in general, as well as in *Apis mellifera* honey bees in particular [105].

### 4.2 Role in caste differentiation

#### 4.2.1 Honey bees

Studies of differences in miRNA expression patterns among eusocial organisms reveal their role in caste differentiation [83]. MiRNAs, in the context of their involvement in the evolution of eusociality, drew attention during the honey bee genome sequencing project, where their caste- and stage-specific expression was demonstrated [106]. Expression levels of various miRNAs differ from worker bees to queens during the pupal stage [27,107,108], thus, leading us to the determining role of miRNAs in caste differentiation.

37 miRNAs were found to be differentially expressed in worker and queen larvae and were associated with the insulin, MAPK, mTOR, and Wnt signaling pathways [108].

In a study conducted by Ashby and colleagues differences in miRNA expression were observed in larvae of drones, queens and workers. Of the 164 miRNAs identified, 120 showed different expression levels between the two castes and 27 showed differences between all three castes. Examination of gene ontology terms revealed that miRNAs preferentially target transcription factors involved in pathways related to neuronal differentiation [109].

It is known that not only endogenous RNAs but exogenous ones included in bees' food are distributed differently. Some experiments show that feeding queen-larvae with royal jelly accompanied with “worker's” miR-184 can essentially affect its future phenotype by lessening body and wing size [85,105].

On the other hand, a recent extensive study has shown that miRNAs contained in the extracellular vesicles of royal jelly enhance anti-apoptotic processes and reduce the expression of pro-apoptotic genes [110].

Differential expression of miRNAs has also been shown within the worker caste in nurses and foragers of *Apis mellifera*. The alterations in miRNA gene expression seen in young nurse bees and older forager bees indicate that miRNAs may play a role in age-related shifts in bee behavior [111].

Additionally, specific miRNAs have been linked to processes like hypopharyngeal gland development in nurse bees and honey processing in foragers. MiR-184 and miR-252a in nurse bees could be linked to the secretion of royal jelly, while decreased levels of miR-11 and miR-281 in foragers contribute to honey processing [112].

There is a large group of miR-279 paralogs that play an important role in eusociality. Their influence has been shown in various models. For example, miR-279a has been shown to potentially play a role in behavioral transition in bees through the regulation of sucrose. Significantly higher expression of miR-279a was found in the brains of nurse bees compared to forager bees, regardless of their age. Overexpression of miR-279a attenuates forager sensitivity to sucrose, whereas its absence enhances it [113]. In addition, miRNA let-7 was found to affect sucrose regulation in gatherers through decreased expression of the dopamine receptor Amdop2 [114].

#### 4.2.2 Ants

Unlike other Hymenoptera, the eusociality of ants is their synapomorphy. Therefore, in order to investigate the mechanisms of sociality in this group, it is necessary to distinguish between unique eusocial traits of ants and parallelisms with other groups. Among ncRNAs, the effects of miRNAs on ant behavior are best studied.

The effect of miRNA on caste membership is more eloquently demonstrated in the ant species *Harpegnathos saltator*. In this case, it is reported that after the queen ant's passing, the worker ants transform into the gamergate caste, which is capable of reproduction, by a global decrease in the expression of some miRNA genes. However, the thorough mechanism remains unknown [115,116].

For the ant *Solenopsis invicta*, another miR-279 paralog miR-279c has been demonstrated to participate in the division of labor through negative regulation of the target gene Rab8A via control of insulin production. In foragers, miR-279c suppressed Rab8A expression, resulting in decreased insulin levels, which led to a behavioral shift towards nursing behavior [117].

Several miRNA are differentially expressed among castes of *Temnothorax rugatulus* ants. While some of them are unique for this ant, several play consistent roles as well in honey bees, proving ideas of the “genetic toolkit”. MiR-927a and miR-12 are involved in ovary development. Worker ants marker miR-133 have not been described as differentially expressed in different bee castes. So, the unique genes hypothesis is confirming as well [118].

The role of some miRNAs in eusociality has been shown in other Hymenoptera (miR-279 and its paralogs), while others are apparently unique to ants. Only few ant models have characterized miRNA transcriptomes, presenting a huge research gap. Evidence of convergent miRNA pathways yet to be described [84]. Novel information on eusociality development could be obtained by comparing the role of the known eusociality influencing miRNA between models, which is discussed below.

#### 4.2.3 Other models

Crucial part of studies is researching facultative eusocial models. Each such model has its own specificity, which may tell us that the transition to eusociality is a gradual process requiring a certain set of prerequisites rather than a specific sequence of actions. Despite the large number of studies of such models [15], almost all of them ignore non-coding RNAs.

Facultatively eusocial sweat bees *Megalopta genalis* and solitary species express more unique miRNAs in the brain than social species [119]. On the other hand, most miRNA are unique for each species, and less are shared. Only miR-305 was expressed in the brains of all eusocial (including facultatively) species. This partially supports the "unique genetics" of the eusociality hypothesis.

In the primitive eusocial bumblebee *Bombus terrestris*, the miRNA miR-6001 was found to be expressed at higher levels in queens than in workers [68]. It is a common miRNA for all Hymenoptera [84] and is not differentially expressed in honey bee larvae [120]. On the other hand, it has recently been found to play a role in the immune response via interaction with circRNA [121]. MiR-6001 combined with other related miRNA influences Wnt, Hippo and Notch signaling pathways [122] and might play a role in caste and eusocial immunity development.

Another important model for eusociality research is bees with selfish behavior [123].Their genetic evidence is related to a single SNP in the thelytoky locus *th* [74] and surely is not common for eusocial development of other species because of the uniqueness of this mutation. On the other hand, it results in differential splicing [124] and expression of gene sets, potentially similar to regulatory changes associated with other mechanisms. Further research of such models is really interesting in understanding eusociality and questions the “point of no return” hypothesis.

### 4.3 Phylogenetic comparisons

Phylogenetic comparisons reveal differences between some miRNA groups and all other Hymenoptera. At the same time, it is difficult to speak about the similarity of these groups in different insects, because their role is not always clear. Assays, provided by qRT-PCR experiments, show that expression of such loci as miR-9a, C3345, and C5152 are specific for worker bees' abdomens, while C1504 and miR-71 are predominantly expressed in queens’ abdomens. In accordance with the authors' hypothesis, differential expression analysis accompanied by computational prediction of potential miRNAs point out that miRNA expression itself is distributed very specifically depending on tissue type, development stage and caste division [107].

Another case of eusociality-related miRNA are genes of miR-279 family (miR-279c and miR-279d) that exhibit another interesting nature of miRNA distribution in honey bees and inheritance features in whole Arthropoda, which were described above in both ants and bees contextes. Functionally, this gene family is thought to be involved in neuronal development, odor sensitivity, and circadian rhythms. Evolutionally, miR-279d was lost in Hymenoptera order, but restored in eusocial Aculeates through duplicates, while miR-279c was developed conservatively in Hymenoptera and subsequently lost in non-social Aculeate species [105]. Authors’ hypothesis demonstrate that not only functional and differential expression studies can be utilitary used in miRNA studies, but also phylogenetic analysis of their inheritance peculiarities in different Hymenoptera species, thus, giving scientists a hint in their biological role in honey bee physiology and development.

Greenberg and colleagues [125] identified 25 miRNAs unique to Hymenoptera, 20 of which are specific to honey bees. They examined the conservation of these 20 miRNAs and found that 19 of the 20 honey bee-specific miRNAs were also present in other eusocial Hymenoptera genomes. In addition, 5 miRNAs were conserved in all eusocial Hymenoptera, but were not found in other species. Most researched miR-184 and miR-279 are common Hymenoptera miRNA, and their presence could be detected in brains of solitary species as well [119], so their differential expression and variants might be crucial. Unique for social species miR-163, 279c, 980 and 3786 claimed to be most influential. These miRNAs were not identified in non-social wasps *Nasonia longicornis* and *N. vitripennis*.

A research conducted on the genome, miRNA, transcriptome, and methylome of the brain of two species of eusocial insects - the wasp (*Polistes canadensis*) and the ant (*Dinoponera quadriceps*) identified four families of miRNA that are specific to hymenopterans, along with an additional nine families that were shared by apocritans. However, the study did not reveal evidence of the role of DNA or miRNAs in the regulation of phenotypic differentiation [29].

Interestingly, only ∼100–200 miRNA could be defined using traditional miRNA-sequencing strategies in bees [76,119]. Among them, 20 is significantly differentially expressed between castes [76]. Crucial part of unique honey bee's miRNA are unique for all eusocial Hymenoptera and can get lost among solitary species. Most researched and universal are miR 184 and miR 279, which is hypothesized to influence similar pathways. So it might be not only adaptation to miRNA presence, but also their usage in real genetic programs. Overall, this confirms the existence of a "genetic toolkit". Nevertheless, future studies should not only look for miRNAs that are shared between eusocial organisms, but also unique and those that they lack.

### 4.4 SiRNA

The processes of RNA interference are well studied in many eukaryotes and have been used as modulators of gene expression and antiviral defense mechanisms. SiRNAs are capable of suppressing the processes of matrix synthesis at transcription or translation stages by degrading the matrix RNA [126,127]. Key difference between work of siRNA and miRNA is in their development - miRNAs are typically formed from harpins, while siRNAs emerges from double-stranded RNAs with full matches.

None of siRNA related to eusociality is predicted. In addition to insufficient study, this may have another reason - misclassification with other small RNA types. Only antiviral response role was reported [128]. Any immune role could be related to eusociality development, so overall study on them is required. Question about differential expression of siRNA between castes might indicate differences in social functions [58,127].

### 4.5 LncRNA

Most lncRNAs are transcribed similarly to mRNAs by RNA Pol II and can be capped, spliced, and polyadenylated [129]. Studies show that lncRNAs have more specific expression patterns than mRNAs and are only abundantly expressed in certain cells or tissues, indicating tighter regulation [130]. They can also be downregulated by miRNAs and can act as a miRNA sponge like circRNAs [131,132]. Other possible mechanisms of lncRNA action include direct binding with DNA [133], alternative splicing mediation [134], decoy of transcription factors [133], protein scaffolding [135] and some others including even polypeptides encoding [136]. However, real mechanisms of lncRNA functioning in Hymenoptera are practically not shown and we have to be guided by the data known for *Drosophila* and other models. Number of predicted lncRNA is much larger than ones with at least predicted targets or functions.

In honey bees, the number of lncRNAs expressed at the adult stage is approximately twofold lower than that of mRNAs [76]. Surprisingly, most of them are not related to any stage. 5 % of them are queen-specific, in contrast to 0.4 % of worker-specific lncRNAs [76]. On the other hand, far fewer lncRNAs are identified in ants [78]. In all Hymenoptera models, there is extensive evidence of the involvement of lncRNAs in gene expression.

#### 4.5.1 Honey bees

List of lncRNA can possibly influence bees' caste development. An in-depth study of honey bees foragers and nurses transcriptome revealed distinct lncRNAs and their possible functions. Moreover, some lncRNAs were found to target the cGMP-dependent protein kinase gene, which plays an important role in regulating the behavioral transition. Additionally, a link was identified with the *acsf2* gene, which is involved in fatty acid metabolism, and vitellogenin signaling. The Wnt pathway is actively involved in this type of signaling. This is fully enriched with lncRNAs and mRNAs targeted with it. The Wnt pathway, in turn, interacts with the JH cascade, thereby influencing bee behavior [137].

For long noncoding RNAs, involvement in signaling communication has also been demonstrated. The differential expression of 9 lncRNAs was observed in the brains of dancing and non-dancing bees. Two of them are likely involved in the waggle dance of honey bees through gene modulation [138].

In honey bees drones, higher expression of KS-1 was observed in several neurons at higher levels compared to queens, implying a role in drone-specific brain functions [139]. AncR-1, has been discovered in the honey bee brain and is also active in reproductive and secretory organs [140]. The localization of AncR-1 and KS-1 transcripts within a specific area of the neuronal nucleus indicates their involvement in distinct neuronal functions in the brain [141]. Comparative analysis of 5 transcriptomes of *Apis cerana* workers bees of different ages (3 foragers and 2 nurses), together with uniquely expressed genes, also revealed differentially expressed lncRNAs, suggesting their involvement in the regulation of behavioral transition [142]. Nb-1 is overexpressed in nurse bees' brains compared to foragers and queens, and has been identified as participating in the regulation of octopamine and juvenile hormone release during the transition from nursing to foraging behavior [143].

Lncov1 and lncov2 were the first to find differentially expressed lncRNA in larval ovaries. Furthermore, the peak of lncov1 expression coincided with the onset of autophagic cell death in the ovaries of worker bee larvae [144]. Subsequently it was shown that lncov1 interacts with the Tudor *Staphylococcus* nuclease (Tudor-SN) protein to promote apoptosis in the ovaries of worker larvae, leading to the development of the worker caste [95,141].

LncRNA could potentially target miRNA as a sponge. It was previously well-described on immune processes, but these interactions include none of eusocial-related ncRNAs [145]. On the other hand, mir-184, miR-14 and other RNAs which are differentially expressed during ovary development have been predicted targeting lncRNA [146]. This mechanism is promising and available now for open access data-mining.

So, lncRNA-dependent gene regulation plays a crucial role in the development and maintenance of eusociality in insects, including caste determination and division of labor. List of potential targets are much bigger in comparison to miRNA. but they are less researched nowadays.

#### 4.5.2 Ants

Comparative study of ants exhibiting different social strategies identified 438 and 359 high-confidence lncRNA gene patterns for *Harpegnathos saltator* and *Camponotus floridanus*, respectively. For *H. saltator*, caste-, brain-, and developmental stage-specific lncRNAs have also been shown [78].

Comparative transcriptomic analysis between virgin and mated queens of the ant *Solenopsis invicta* [147] revealed 295 mRNA genes and 65 lncRNA genes exhibited differential expression levels. Of these, 17 lncRNAs were highly expressed in virgin queens, while 47 lncRNAs were highly expressed in mated queens. Moreover, some lncRNAs were found to interact with specific coding genes, like hexamerin 1, suggesting a potential role in regulating brain gene expression during reproductive transition. Interestingly, ant reveal much lower distribution of lncRNA in comparison to honey bees, indicating different evolutionary strategies of different Hymenoptera branches. The difference in lncRNA abundance can be explained by different trends in genome function, including the function of other ncRNAs.

### 4.6 PiRNA

PiRNAs, or PIWI-associated RNAs are encoded by loci or clusters of the same name and represent a short (∼21–32 bp) single-stranded RNAs synthesized from single-stranded piRNA-precursors [148]. Biogenesis mechanisms are still discussed, but main is supposed to be “ping-pong” mechanism (Fig. 1) [149]. Functionally, piRNAs manage genomic stability throughout repression of repetitive transposable elements and discrete genes [150,151].

Hymenoptera exhibit a considerable degree of variability in the number of mobile genetic elements [152]. However, it does not show a direct connection with eusociality [153].In *Drosophila* flies, a mechanism related to piRNA expression has been observed to result in hybrid dysgenesis when active transposons are inherited through the paternal lineage [154]. We hypothesize that the conservation of such a mechanism in Hymenoptera will lead to stronger selection for active transposons - violations of piRNA expression will be immediately eliminated in the male (haploid) lineage. Theoretically, this can explain two known facts: in addition to the reduced number of repeats and noncoding elements in Hymenoptera [152], many noncoding DNAs are actively expressed, which is possible only in the absence of their inclusion in the piwi-locus and, at the same time, inactivity of mobile genetic elements.

This hypothesis is indirectly supported by articles on honey bees and ants [97,155]. Drones showed higher piRNA levels in contrast to queens and workers [97]. Male's reproductive system contains fewer amounts of piRNAs than female's or egg's [96], but a bigger variety [97]. In contrast, solitary bumblebees males semen contains little piRNA with similar and only slightly higher transposon level [156]. It might indicate another transposon regulation mechanism and a potential role of piRNA in eusociality. Similarly, ants also have expression of piRNA in queens ovaries [118], but it might be similar to solitary species.

PiRNA also could play an immune role. It was reported that piRNA are involved in antifungal response in *A. mellifera* larvae [157]. Experimental data demonstrate that inoculated or PIWI-depressed organisms show high replicability of pathogens, whether it is fungi or viruses, thus indicating an important role of piRNAs in immunological response. Some articles reported no viral-related piRNA in honey and bumblebees [158,159]. But their genomes often contain endogenous viral elements (EVEs), which could possibly produce antiviral piwiRNAs [156,160].

Another interesting fact of piRNA participation in gene regulation is through silencing some nontransposable lncRNAs [97]. Embryonic localization of this regulatory circuit can be a very promising feature in further investigation of potential piRNA-lncRNA-associated caste determination in eusocial species.

PiRNAs unambiguously play an important role in the evolution of Hymenoptera in general. The comparison of piRNA targets between social and solitary species may help to delineate their specific functions. Interestingly, unlike other species, piRNA expression in Hymenoptera is not restricted to ovarian and embryonal development [97,155]. Therefore, piRNAs may play a key role in the maternal heterochrony [15] mechanism. The lack of comparative datasets of small RNA expression currently limits the understanding of their role.

### 4.7 CircRNA

In eukaryotic organisms, evidence of the important role of circular RNAs (circRNA, ciRNA) in the regulation of gene expression is only relatively recent. CircRNAs are formed as a result of mRNA splicing. Most of them contain only exons; other types are less common. As a result of a process known as direct back-splicing, complex RNAs are formed. Most often, this results in non-functional mRNAs. Some of them, unlike most other ncRNAs, could be potentially encoded in the mitochondrial genome (mecciRNAs) [161]. However, these findings were not yet discovered in Hymenoptera neither in *Drosophila* nor in any insects, while their roles might be crucial.

The modes of action of circRNA in some cases are similar to lncRNAs. For example, they can influence transcription via alternative splicing regulation [162,163], direct protein activation [163,164], protein scaffolding [165] and others [162,[164], [165], [166]]. One of the most common mechanisms of circRNA action is polymerase activation at circRNA's own expression sites. Another important mechanism of circRNA is operation as a small RNA sponges. Finally, circRNAs with IRES can function as mRNAs [162], but this has not previously been shown in Hymenoptera. In general, circRNA plays a role in the immune response regulation [121], thus being an important marker of immune activation.

Number of expressed circRNA in honey bees is near to mRNA. This can be explained by the coupled mechanism of biogenesis. In contrast, while only ∼2 % of mRNA are caste-specific in honey bees, among circRNA 38 % are specific to workers, and 5–12 % are unique to queens. Most of them contain only exons, less - exon-introns. Other (and considered as main functional) types present less than 10 % of all diversity [76].

In insects it was found that a whole cascade of circRNA-mediated reactions is triggered in response to the pathogen. There are different variants of circRNA action, depending on whether the pathogen is fungal, viral or bacterial. As a rule, all scenarios are aimed at the activation of MAPK, Toll or Imd signaling pathways that trigger phagocytosis, acceleration of proliferation and proteolysis processes [167,168]. A number of circRNA are differentially expressed between diseased and healthy honey bees, indicating their role in the immune response [121,169]. Recent studies have revealed that circRNAs (circ-5197 and circ-16640) competitively regulate some miRNAs in honey bees, thereby affecting reproductive functions, as reminded previously [169]. There is also evidence for the potential involvement of circRNA in midgut morphogenesis in worker honey bees, through participation in various molecular signaling. The main mechanism in this case is supposed to be through targeting miRNAs [170]. Also, a pair of circ-2015 and miR-14-3p claimed to be interacting. CircRNA sponge influences egg-laying in honey bees [171]. Another absorbing circRNA target is mir-6001 [121], which was described above.

Through RNA-Seq with exonuclease enrichment, Thölken et al. detected multiple circular RNAs in the brains of honey bee nurse and forager bees. Notable differences in expression were seen for circRNAs circ-1780 and circ-1822 between the two groups. circ-1822 is more expressed in forager bees' brains compared to nurse bees, with this distinction likely linked to age rather than tasks. On the other hand, circ-1780 displays higher expression in nurse bee brains, irrespective of age-related task changes [172].

In general, the already available results concerning the study of circRNA in the regulation of gene expression suggest a role of this transcript in gene expression processes (morphogenesis). Such mechanisms can directly influence the eusocial behavior of Hymenoptera. Studies on other models will allow phylogenetic comparisons. CircRNAs look promising also because of their connection with immunity and may be involved in the formation of the "point of no return".

### 4.8 Environmental RNA

As described above, environmental RNAs (envRNA) may also play an important role in caste differentiation and the development of eusociality together. It is worth noting that although envRNA is believed to be unstable, mechanisms for its stabilization are known *in natura*. Small RNAs are often more stable (or longer RNAs simply collapse to smaller ones), so major interactions are carried out via miRNAs and dsRNA [173,174]. The pathways of envRNA production and transmission are under investigation, but it is clear that they co-evolve in symbiotic organisms and are likely differentially expressed [173,175].

A key issue in envRNA functions in eusociality is their uniqueness, tropism, and processing. Eusocial species gain attachment to a specific habitat (as do many solitary species), which enables envRNAs to co-evolve efficiently [175,176]. However, it is likely that all species in consortia have a similar environmental RNAi background. But how similar is it within a single eusocial colony, where different castes differ in ecology?

EnvRNA can be transmitted through the gut into hemolymph and then into worker and royal jelly in binding with proteins [177]. Their diversity is distinct [178], so different envRNAs are passed between different castes. Classifiable sequences originated from plants and fungi to a greater extent than from bacteria and bees [178]. The dsRNAs represented 10–35 % of all sequences. All environmental RNA possibly influence caste development [110] and microbiome composition [179].

Plant miRNAs in beebread can slow down honey bee development and reduce their body and ovary size, ultimately preventing larvae from becoming queens and instead developing into worker bees [180]. The amTOR gene, involved in caste differentiation [181,182], is a direct target of the plant miR162a found in beebread [183]. Its effect on bee development is the same as that on *Drosophila* [180], by reducing body and ovary size. We hypothesize that if in solitary species it should be eliminated by negative selection, in social species it may lead to the emergence of disruptive selection and caste formation.

EnvRNA-related mechanisms might be similar across all eusocial organisms. Surprisingly, there is no research on social models except bees, presenting a huge research gap.

### 4.9 Other types

In addition to the types of non-coding RNAs described above, there are other types of non-coding RNAs. The existence of a role for housekeeping RNA in eusociality is rather difficult to assume, although snRNA and snoRNA, for example, can be transmitted and constitute major parts of worker and royal jelly [180]. Some potentially functional types (scaRNAs) have been identified in other insects [184], and their role in eusociality remains to be explored. Enhancer RNAs represent a special example - their function at the level of histone methylation is known [185], but they themselves are hardly studied.

Viruses, like any pathogen, are very important in the establishment of sociality. Differences in pandemic patterns affect immunity and selection. Viruses themselves become antisense RNA targets, and their RNA itself can trigger RNA interference, fighting immunity. According to the concept of superorganism, the death of a particular organism from infection may be more advantageous for society than its survival.

Viruses, like any pathogen, are very important in the establishment of sociality, affecting immunity and selection [186]. DNA of viral origin incorporated into the genome (EVE) can produce vpiRNA. CircRNAs of host and viral origin are very important in their interaction [187]. Viruses themselves become antisense RNA targets, and their RNA itself can trigger RNA interference by fighting immunity [58,59,127]. For example, some honey bee viruses produce siRNA targeting antiviral immunity RNAs [128]. According to the concept of superorganism, the death of a particular organism from infection may be more advantageous for the society than its survival [58,188]. So, we can expect differences in the associated molecular mechanisms as well - for example, related to anti-vsiRNA circRNA-sponges. In general, pathogen and predator control mechanisms may be similar in all eusocial organisms and form the molecular basis of the "point of no return".

## 5 Conclusions

In conclusion, eusociality represents a remarkable form of social organization observed in various animal groups. The evolution of eusociality is shaped by a complex interplay of genetic and ecological factors. Genetic studies have identified specific genes and regulatory mechanisms associated with reproductive division of labor, caste determination, and social behavior in eusocial organisms.

If we compare the role of different RNAs in the establishment of eusociality, a clear skewness in the studied sites and types of non-coding RNAs becomes apparent. The most studied area is miRNA in the caste development of honey bees. Despite their small diversity, many of them are differentially expressed. Their numbers are close in different Hymenoptera, but only ∼10 % overlap in all. Searches for potential markers of eusociality at the level of presence in the genome are not very successful and identify only one miRNA. The diversity of other types of non-coding RNAs is either poorly studied not in bees or varies greatly between groups. For example, the difference in lncRNA diversity between bees and ants is an order of magnitude, and there is virtually no data for the other classes. It is worth noting that many noncoding RNAs in Hymenoptera have unique expression patterns over time and tissues compared to other insects. For example, piRNAs are expressed much more widely, showing one reason why the non-coding genome is so low in Hymenoptera, and potentially taking on other functions directly related to development. Finally, it is not the phylogenetic gains of eusocial species but their losses that have been virtually unstudied. If convergent evolution follows a path of molecular parallelism, then one would expect it to follow the most parsimonious paths. Theoretically, loss of functions is a more likely event than their acquisition. However, this requires more research.

Various eusociality development theories were discussed. Many ncRNAs may prove to be the molecular basis of the "point of no return" directly or through eusocial immunity. MiRNAs demonstrate convergent evolution in different Hymenoptera clades, offering a genetic toolkit to eusociality. Other types, like some miRNAs, are important in caste development and eusocial behavior, influencing eusocial pathways but being unique to different organisms. Thus, understanding the role of ncRNAs is important for understanding current hypotheses of eusocial development.

In summary, noncoding RNAs play a pivotal role in the emergence and evolution of eusociality in hymenopteran insects. Through their regulatory functions, noncoding RNAs modulate gene expression patterns that are crucial for the development of caste systems, division of labor, and social behavior in eusocial species. The enrichment of regulatory elements such as miRNAs and circRNA in the genomes of eusocial insects underscores the importance of noncoding RNAs in orchestrating the complex genetic networks that underlie eusocial traits.

Moreover, the conservation of these regulatory elements across different eusocial lineages suggests a common genetic basis for eusociality, while the diversity of eusocial insect genomes implies a degree of evolutionary flexibility in the genetic regulation of social behavior. Future research focusing on the interplay between noncoding RNAs, gene regulation, and eusocial phenotypes will further enhance our understanding of the molecular mechanisms that govern social evolution in hymenopteran insects. These findings have broader implications for the study of social evolution and gene regulation, paving the way for advancements in the field of sociogenomics and epigenetics.

## CRediT authorship contribution statement

**Egor Lebedev:** Writing – review & editing, Writing – original draft, Software, Conceptualization. **Daniil Smutin:** Writing – review & editing, Writing – original draft, Validation, Supervision, Formal analysis, Data curation, Conceptualization. **Pavel Timkin:** Writing – original draft, Formal analysis. **Danil Kotelnikov:** Writing – review & editing, Writing – original draft, Formal analysis, Data curation. **Amir Taldaev:** Writing – review & editing, Writing – original draft, Formal analysis, Data curation. **Nick Panushev:** Writing – review & editing, Writing – original draft, Formal analysis, Data curation. **Leonid Adonin:** Writing – review & editing, Writing – original draft, Validation, Supervision, Conceptualization.

## Funding

This work was financed by the Ministry of Science and Higher Education of the Russian Federation within the framework of state support for the creation and development of World-Class Research Centers ‘Digital Biodesign and Personalized Healthcare’ (No 075-15-2022-305).

## Declaration of competing interest

The authors declare no conflict of interest. The funders had no role in the design of the study; in the collection, analyses, or interpretation of data; in the writing of the manuscript, or in the decision to publish the results.
